# Correlation of INHBA Overexpression with Pathological Features, Antitumor Immune Response and Clinical Prognosis in Cervical Cancer

**DOI:** 10.3390/medicina59030495

**Published:** 2023-03-02

**Authors:** Lin Zeng, Xingwang Sun

**Affiliations:** 1 Department of Pathology, The Affiliated Hospital of Southwest Medical University, Luzhou 646000, China; 2 Department of Pathology, The Third Hospital of Mianyang, Sichuan Mental Health Center, Mianyang 621000, China

**Keywords:** cervical cancer, INHBA, pathological features, antitumor immune response, clinical prognosis, correlation

## Abstract

*Background and Objectives*: Cervical cancer (CC) is a malignant tumor occurring in the cervical epithelium, which is one of the most common cancer-caused deaths in females. Inhibin β A (INHBA) is the most widely expressed biomarker of the transforming growth factor-β (TGF-β) family in tumor cells, and has predictive value for tumor development and prognosis. In this study, the expression of INHBA in CC tissue was examined to analyze the relationship between INHBA expression and pathological characteristics, anti-tumor immune response and clinical prognosis of CC. In addition, the factors affecting the prognosis of CC patients were explored. *Materials and Methods*: 84 patients with CC, who underwent surgical resection in our hospital from March 2016 to August 2017, were retrospectively picked. The tumor tissues and normal adjacent tissues of patients with CC were collected, and the expression of INHBA in CC tissues and adjacent tissues was detected using immunohistochemistry (IHC). The relationship between INHBA expression and clinicopathological characteristics of CC patients was analyzed. The relationship between INHBA expression and clinical prognosis was analyzed using the Kaplan–Meier (K–M) survival curve. The levels of anti-tumor immune-response-related factors (interferon-γ (IFN-γ), interleukin-10 (IL-10), tumor necrosis factor- α (TNF-α) and IL-2) were evaluated in patients with negative and positive expressions of INHBA. The patients were followed up for 60 months and were graded as a good prognosis group and poor prognosis group according to whether the patients died or had recurrence and metastasis. Relevant factors affecting the prognosis of the patients were analyzed. *Results:* INHBA was localized in the cytoplasm of cancer tissues. The positive expression rate in cancer tissues was 67.86%, which was much higher than the 28.57% in normal adjacent tissues (*p* < 0.05). Expression of INHBA was closely correlated with Federation of Gynecology and Obstetrics (FIGO) staging, differentiation and lymph node metastasis (*p* < 0.05). Compared with INHBA-negative expression group, the contents of IFN-γ, TNF-α and IL-2 were much lower, while the level of IL-10 was strongly elevated in the INHBA-positive expression group (*p* < 0.01). Eighty-four patients with CC were followed up for 36 months. The K–M survival curve showed that the patients with a positive expression of INHBA had a significantly shorter survival period than the patients with a negative expression of INHBA (*p* < 0.05). There were significant differences in FIGO staging, differentiation, lymph node metastasis and INHBA expression between patients with a good prognosis and poor prognosis (*p* < 0.05). Logistic regression analysis showed that FIGO stage, differentiation degree, lymph node metastasis and INHBA were the factors influencing the poor prognosis of patients with CC (*p* < 0.05). *Conclusion:* The abnormally high expression of INHBA in patients with CC was related to the pathological characteristics, anti-tumor immune response and survival time, and leaded to a poor prognosis. It was speculated that INHBA exerted an important reference role in tumor invasion and clinical prognosis evaluation, which could act as a new target for anti-tumor treatment of CC.

## 1. Introduction

Cervical cancer (CC) is not only the main cause of female cancer deaths in developing countries, but also one of the main causes of female deaths worldwide. According to the relevant statistics, the new annual incidence rate of CC exceeds 530,000 and the number of deaths exceeds 270,000 globally, which seriously affects the lives of patients [1]. Surgical resection is an important method for the treatment of CC, but the recurrence rate of patients after surgery is high and the prognosis is poor. Individualized comprehensive therapy and molecularly targeted drug therapy will be the main directions of clinical research on CC in the future. However, due to the large side effects, low sensitivity, rapid occurrence of drug resistance and other reasons, the 5-year survival of patients with advanced CC is still less than 40%. It may be that pathological tumor interstitial invasion and lymphoid metastasis are risk factors for the development and prognosis of CC [2]. Although great progress has been made in the study of CC invasion and metastasis in recent years, the exact molecular mechanism still needs further study. Therefore, it is important to explore the pathogenesis of CC and find a suitable prognosis target for the clinical treatment of CC.

Inhibin beta A (INHBA) is a heterodimer composed of α and βA subunits and is also a member of the transforming growth factor superfamily. INHBA forms a disulfide-linked homodimer called activin a, which is originally described in 1978 for its role in the hypothalamic–pituitary–gonadal axis [3]. Several studies found that abnormal expression of INHBA exerted various biological functions in different tumor development processes. For instance, the inhibitory role of INHBA in the growth of lung cancer and head and neck squamous cell carcinoma is not obvious, but it enhances the invasive ability of tumor cells, which involves many processes of the body and embryonic development, including cell growth, differentiation, apoptosis, homeostasis and so on [4]. The expression level of INHBA in various malignant tumor tissues, such as gastric cancer and breast cancer, is significantly increased. Its expression level is obviously related to the patient’s condition, and INHBA-negative patients have a better overall survival and progression-free survival, which indicates that INHBA may be used as an independent prognostic factor for multiple cancers [5]. Importantly, high expression of INHBA is associated with tumorigenesis in women. High expression of INHBA in ovarian cancer tissues is associated with an increased risk of death from high-grade serous ovarian cancer, knockout of the INHBA gene reduces the activation of ovarian cancer stromal fibroblasts and inhibits the growth of tumor cells, and upregulation of the INHBA gene induces stromal fibroblast activation to support tumorigenesis [6]. In breast cancer cells, downregulation of INHBA can delay the growth of primary tumors, inhibit the migration of tumor cells, and reduce the possibility of lung metastasis [7]. At present, it has been found that there is differential expression of INHBA in cervical cancer tissues [8], but whether it is related to the response of cervical cancer immune cells is unclear.

In this study, 84 patients with CC, who underwent surgical resection in our hospital from March 2016 to August 2017, were picked. The expression of INHBA in CC tissue was examined to analyze the relationship between INHBA expression and pathological characteristics, anti-tumor immune response and clinical prognosis of CC, and explore factors affecting the prognosis of CC patients.

## 2. Materials and Methods

### 2.1. General Materials

**Figure 1:**
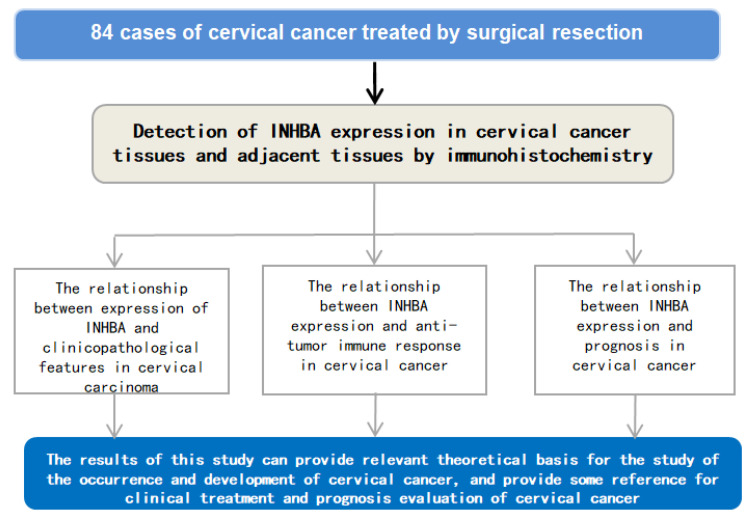
The experimental process. INHBA, inhibin beta A.

Eighty-four patients with CC, who underwent surgical resection in our hospital from March 2016 to August 2017, were retrospectively picked. The tumor tissues and normal adjacent tissues of patients with CC were collected. Cervical cancer tissue was derived from patients who underwent total hysterectomy and cervical conization for cervical lesions. The age range of the patients was 25–68 years old, and the average age was 45.23 (±2.85) years. Inclusion criteria: (1) All subjects were diagnosed with CC by pathological examination; (2) The patients and their families signed an informed consent form; (3) Patients underwent surgical resection; (4) Patients did not receive radiotherapy and chemotherapy before participating in the study; (5) None of the patients received chemotherapy, radiotherapy, endocrine therapy, or biological therapy before surgery. Exclusion criteria: (1) Patients with distant metastasis before participating in the study; (2) Patients with mental-related disease history and a disability to cooperate well with the research; (3) Patients combined with other malignant tumors; (4) Patients with incomplete clinical medical records; (5) Patients during the pregnant or lactating period. This study was ratified by the hospital Ethics Committee and conformed by medical ethics (Approval number: No.KY2022049). The experimental process is exhibited in Figure 1.

### 2.2. Immunohistochemistry (IHC) [9]

The expression of INHBA in CC tissues and adjacent tissues was detected using IHC. The execution was carried out according to the following description: CC tissues were fixed into paraformaldehyde overnight. The appropriate size tissue was placed in the cassette and dehydrated in the following order: 75% alcohol for 1.5 hours (h), 95% alcohol for 1.5 h, 95% alcohol for 1 h, absolute ethanol for 1.5 h, absolute ethanol for 1 h, xylene (no. 1) for 0.5 h, xylene (no. 2) for 0.5 h. The embedding machine was opened, and then the cold table, cold spot, paraffin tank, and tissue tank, respectively, for work. Then, the paraffin block was loaded with an iron lunch box and put into the tissue tank of the embedding machine to heat and dissolve. The dehydrated tissue, together with the embedding box, was put into the machine tissue tank in turn, and then put into the paraffin lunch box (no. 1) for the second wax immersion, and then paraffin lunch box (no. 2) for the third wax immersion. The microtome was turned on, the wax block fixed, and the distance between the blade and the wax block adjusted. Then, embedded tissues were consecutively cut for four specimens, with a thickness of approximately 3–4 μm. The sections were placed on a sterile clear glass slide, and the temperature of the incubator was set to 60 °C, and dried for about 1 h. A phosphate buffer solution (PBS) was used as primary antibody in the negative control group. Slices were performed with dewaxing and hydration at room temperature in the following order: xylene (no. 1) for 10 min, xylene (no. 2) for 8 min, xylene (no. 3) for 8 min, absolute ethanol for 5 min, 90% ethanol for 3 min, 80% ethanol for 3 min, 70% ethanol for 3 min, distilled water for 3 min three times and PBS for 3 min three times. Subsequently, the slices were added to the citric acid antigen retrieval solution for antigen retrieval for elixation for 20 min, and then stood and cooled for 20 min. An appropriate amount of PBS solution was removed for rinsing. The tissues were removed, the water was shaken off, and the water droplets were absorbed using an absorbent. The tissue was circled with a histographic stroke with a complete circle. The hydrogen peroxide solution was dropped on the sections to block endogenous peroxidase activity. The sections were incubated with INHBA antibody at 4 °C overnight, and the sections were incubated with horseradish-peroxidase-labeled secondary antibody at 37 °C for 15 min, after washing. A color-developing solution was added for color development. After about 5 min, the slices were thoroughly rinsed with tap water and counterstained with hematoxylin. Next, the sections were subjected to conventional gradient anhydrous alcohol dehydration, xylene transparent, and neutral resin sealing. The positive criteria for the section staining were brownish-yellow or brown staining of the cells. The granules with positive expression of INHBA were located in the cytoplasm and a few in the nucleus. Four representative high magnification visual fields were selected for each sample. Two hundred tumor cells were counted in each sample, and the average value was taken. According to the percentage of the colored cells in the counted cells, the number of positive cells divided into less than 10% was 0, the number of positive cells between 10% and 25% was 1, the number of positive cells between 26% and 50% was 2, the number of positive neurons between 51% and 75% was 3, and the number of positive cells greater than 75% was 4. The number of positive cells <10% was negative expression. The pathological staining results were blinded by two pathologists without knowing the clinical data of the samples, and the examination results were evaluated and scored twice. If any dispute arose, the researchers consulted with each other and a comprehensive judgment was performed.

### 2.3. Outcome Measures

According to the expression of INHBA, patients with CC were divided into INHBA-negative expression group (57 cases) and positive expression group (27 cases), and the serum of the patients was collected. The contents of anti-tumor immune-response-related factors (interferon-γ (IFN-γ), interleukin-10 (IL-10), tumor necrosis factor-α (TNF-α) and IL-2) were evaluated by ELISA. The steps were as follows: 50 μL standard sample and diluted sample to be tested were added into the reaction hole, respectively, and then 50 μL Biotin-labeled antibody was added immediately. The membrane plate was covered and shaken gently, and incubated at 37 °C for 60 min. The liquid in the hole was shaken off, and each hole was filled with the detergent. The plate was shaken and patted to dry with absorbent paper. Eighty microliters of affinity enzyme HRP was added in each hole, gently shaken and incubated at 37 °C for 30 min. Fifty microliters of Matrix A and B were added in each hole, gently shaken and incubated at 37 °C for 10 min without light. The enzyme standard plate was taken out and quickly added with 50 μL terminate solution to each hole. The OD value was immediately read at 450 nm after adding the termination solution on the microplate reader.

Follow-up: The follow-up was continued for 60 months after treatment. The first follow-up was conducted one month after the treatment, and then with every three months by telephone or outpatient contact. The endpoint was death, distant metastasis, local recurrence or an increased tumor stage. At the end of the follow-up, the patients with a poor prognosis, such as death, distant metastasis, tumor progression and local recurrence, were divided into the poor prognosis group, and the other patients were divided into the good prognosis group. The clinical data of patients, including age (≥50 years old, <50 years old), Federation of Gynecology and Obstetrics (FIGO) stage (stage I, stage II), tumor size (>3 cm, ≤3 cm), differentiation degree (low differentiation, medium differentiation and high differentiation), number of pregnancies (>3 times, ≤3 times), histological type (adenocarcinoma, squamous cell carcinoma and other types), depth of invasion (T1, T2, T3 and T4), and lymph node metastasis (yes or no), was collected, and the related factors affecting the prognosis of the patients were analyzed.

### 2.4. Statistical Analysis

The enumeration data, such as the positive expression rate of INHBA and age, were expressed in (cases (%)) and compared using an χ^2^ test. The relationship between INHBA expression and the clinical prognosis of patients with CC was analyzed by a K–M survival curve. The influencing factors of the patients’ prognosis were analyzed using a logistic regression curve. The SPSS 24.0 software (IBM Corp., Armonk, NY, USA) was used for statistical data analysis, and the difference was considered statistically significant when *p* < 0.05. 

## 3. Results

### 3.1. Detection of INHBA Expression in CC Tissues by IHC

**Figure 2:**
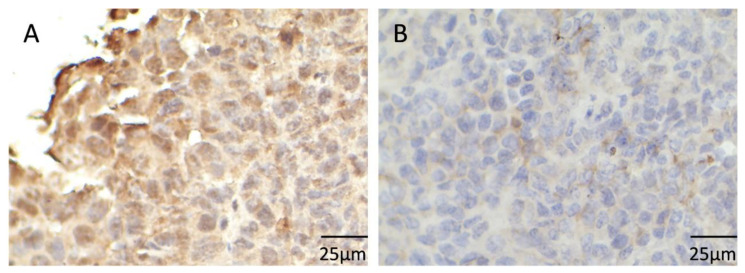
Detection of inhibin beta A (INHBA) expression in cervical cancer (CC) tissues by immunohistochemistry (IHC) (×400). (**A**) Positive INHBA expression; (**B**) Negative INHBA expression.

The expression of INHBA in CC tissues and adjacent tissues was detected by using IHC. INHBA was localized in the cytoplasm of cancer tissues. The positive expression rate in cancer tissues was 67.86%, which was much higher than 28.57% in normal adjacent tissues (*p* < 0.05, Table 1 and Figure 2). These results suggested that INHBA was abnormally expressed in CC tissue, and the positive expression of INHBA was closely related to the occurrence and development of CC.

### 3.2. The Relationship between Expression of INHBA and Clinicopathological Features in CC

The relationship between the expression of INHBA and the clinicopathological characteristics of patients with CC was further analyzed. The expression of INHBA was closely correlated with FIGO stage, differentiation and lymph node metastasis (*p* < 0.05), but not with age, tumor size, number of pregnancies, histological type and depth of invasion (*p* > 0.05, Table 2).

### 3.3. The Relationship between INHBA Expression and the Anti-Tumor Immune Response in CC

**Figure 3:**
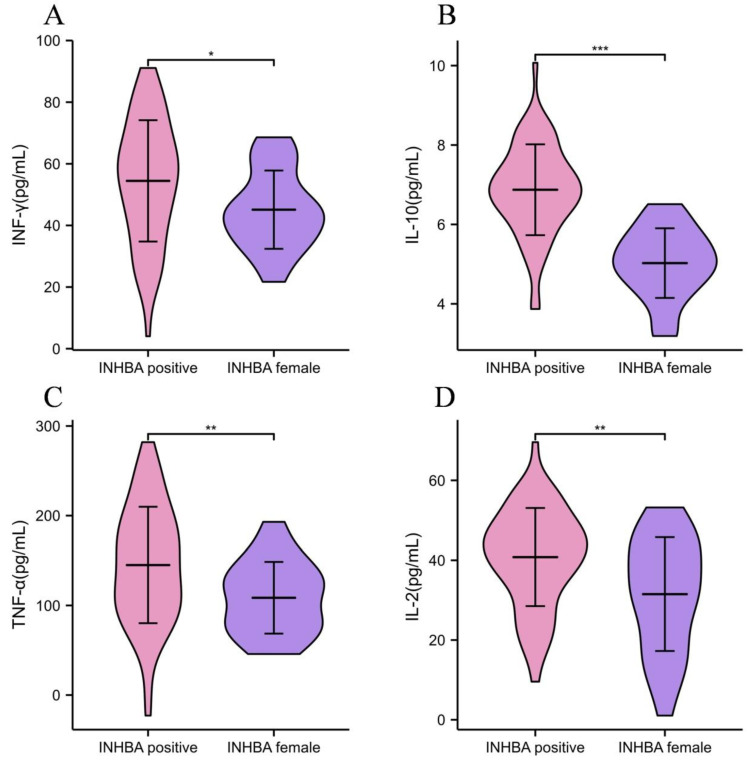
Comparison of anti-tumor immune response indexes in patients with different INHBA expressions. (**A**) IFN-γ content of patients with different INHBA expression; (**B**) IL-10 level in patients with different INHBA expression; (**C**) TNF-α content in patients with different INHBA expression; (**D**) IL-2 level in patients with different INHBA expression. * *p* < 0.05, ** *p* < 0.01 and *** *p* < 0.001.

The relationship between the INHBA expression and the anti-tumor immune response indicators in patients with CC was further analyzed. Compared with INHBA-negative expression group, the contents of IFN-γ, TNF-α and IL-2 were significantly reduced and the content of IL-10 was strongly elevated in the INHBA-positive expression group (*p* < 0.01, Table 3 and Figure 3). The results showed that the positive expression of INHBA was related to the tumor immune infiltration in patients with CC.

### 3.4. The Relationship between INHBA Expression and Clinical Prognosis of CC

**Figure 4:**
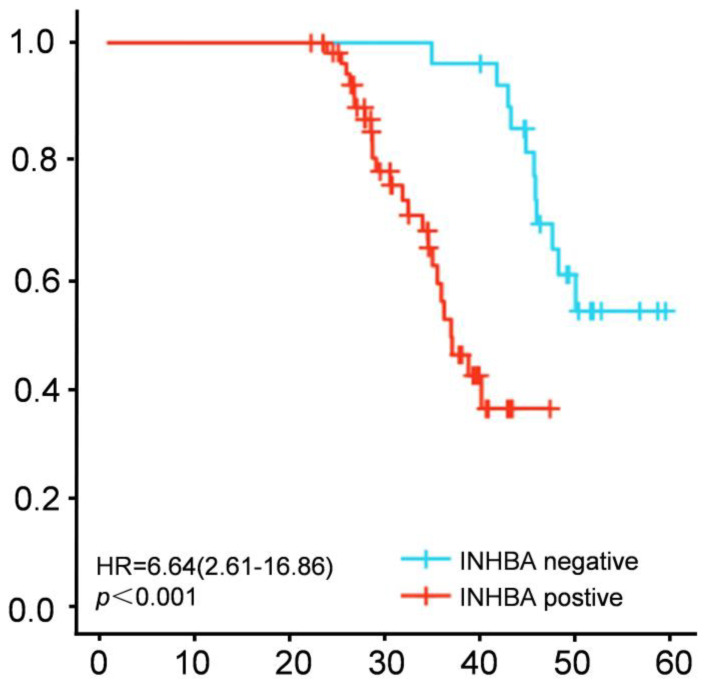
The relationship between the INHBA expression and the clinical prognosis of patients with CC was analyzed through the Kaplan–Meier survival curve.

To explore the effect of the INHBA expression on the survival time of patients with CC, 84 patients with CC included in the study were followed up, with a median follow-up time of 25 months (5–60 months). The Kaplan–Meier survival curve showed that the patients with a positive expression of INHBA had a significantly shorter total survival rate than the patients with a negative expression of INHBA (*p* < 0.05, Figure 4). The above results displayed that the positive expression of INHBA was related to the poor prognosis of patients with CC.

### 3.5. Univariate Analysis of Clinical Prognosis

A significant difference was observed in FIGO staging, differentiation, lymph node metastasis and INHBA expression between patients with a good prognosis and poor prognosis (*p* < 0.05). However, there were no significant difference in age, tumor size, number of pregnancies, histological type and depth of invasion between the two groups (*p* > 0.05, Table 4). The results indicated that INHBA expression might affect the progression and prognosis of CC.

### 3.6. Multivariate Analysis of Clinical Prognosis

Logistic regression analysis showed that FIGO stage, differentiation degree, lymph node metastasis and INHBA were factors influencing the poor prognosis of patients with CC (*p* < 0.05, Table 5). The results indicated that the level of INHBA was an independent risk factor for the prognosis of patients with CC, and INHBA might be a molecular marker for the prognosis of patients with CC.

## 4. Discussion

CC is the fourth most common cancer among women worldwide, after breast, colorectal and lung cancers. With the change in people’s lifestyles and the increasing pressure, the incidence rate of CC has increased year by year, which seriously affects the life of patients. Surgery combined with radiotherapy and chemotherapy is an important method for CC treatment, but postoperative recurrence is still an important reason for the poor prognosis of patients with CC [10]. Local invasion and metastasis of tumor cells are important ways of tumor progression, during which normal cells will be transformed into cancer cells due to biological factors, physical factors and chemical factors, and cell infiltration can establish a new base in distant organs through blood flow, to form distant metastasis. Aggressiveness in tumor development depends primarily on complex biochemical and biological alterations in the tumor cells themselves and the associated matrix [10]. However, the current clinical understanding of the mechanism of tumor invasion and metastasis is still very limited, and further elucidating of the targeted regulation mechanism of tumor cells will provide new ideas for the treatment of CC. Therefore, searching for reliable genetic molecular and immunohistochemical markers for the early diagnosis and evaluation of the patient prognosis is also a momentous task of modern oncology. 

Currently, research on the progress and mechanism of CC mainly focuses on DNA repair, immune escape mechanism and so on. INHBA is a protein-coding gene, belonging to transforming growth factor β superfamily, which may play a considerable role in the progression of various cancers. INHBA silencing inhibited the proliferation of nasopharyngeal carcinoma cells and the invasion of SUNE1 [11]. It has been reported that INHBA expression detected in gastric tumor tissue has increased at least twofold compared to adjacent normal tissue, and INHBA gene silencing is able to inhibit gastric cancer cell migration and invasion through the transforming growth factor β signaling pathway [12]. In addition, INHBA has been proven to be a prognostic factor for patients with colon adenocarcinoma, and the expression of INHBA in colon cancer is significantly correlated with the stage of tumor lymph node metastasis (TNM) [13]. However, the role of INHBA in tumorigenesis and metastasis of CC remains to be elucidated. In this study, the content of INHBA was much higher in CC tissues compared with normal adjacent tissues, and the positive expression of INHBA was closely related to the clinical prognosis of patients with CC. It has been reported that the expression of INHBA is upregulated in colorectal cancer tissues, which is strongly related to vascular cancer thrombus, vascular wall invasion and lymph node metastasis, suggesting that a high expression of INHBA may mainly participate in the evolution of colon cancer [14]. Therefore, the level of INHBA is significantly increased in malignant tumors, such as gastric cancer, nasopharyngeal cancer, colorectal cancer and cervical cancer, and its high expression is closely related to the poor prognosis of patients.

The immune response plays an important role in maintaining normal physiological function and disease progression. IFN-γ, IL-10, TNF-α and IL-2 are important indicators of immune function. Therein, IFN-γ is a unique member of the IFN family. Natural killer cells and natural killer T cells are the main production cells of IFN-γ in innate immunity. IFN-γ not only exerts a key role in coordinating innate and adaptive immune responses against viruses, bacteria and tumors, but has an important role in promoting pathological inflammation [15]. A previous study has revealed that the levels of IFN-γ, IL-4 and IL-6 in the serum of patients with CC are significantly higher than that in the normal population, which may play an important role in the occurrence of CC [16]. In addition, IFN-γ may promote the secretion of cytokines such as IL-1β, TNF-α, and IL-12, and trigger Th1 antigen-specific reaction and immune response, thus enhancing immune function. The inhibition of IFN-γ signal transduction can reduce the interferon-stimulating gene in cancer cells, which exerts a certain anti-tumor role [17]. IL-2 is a pleiotropic cytokine mainly produced by antigen-stimulated CD4+T lymphocytes, which has as an important role in promoting a cellular immune response against intracellular microorganisms and cancer cells [18]. Some scholars have found that the level of IL-2 in the vaginal fluid of the cervical lesion patients with different lesion levels is significantly different from that in healthy women, and same results are also found before and after treatment, which indicates that the recovery of humoral immune function may promote the outcome of patients with cervical lesions [19]. IL-10 is an anti-inflammatory cytokine that can act as a negative regulator of the immune response to microbial antigens. T-cell failure is one of the main obstacles in cancer immunotherapy. It is reported that IL-10 gene polymorphisms play a protective role in the development of CC, which may play a role in immunosuppression to a certain extent [20]. INHBA is significantly associated with immune infiltration, especially T cells, which means that INHBA may be involved in tumor immunomodulation. The recruitment of immunosuppressive cells (i.e., Tregs) is known to be a key process leading to an immune evasion [21]. The results of this study showed that the contents of IFN-γ, TNF-α and IL-2 were sharply decreased, and the level of IL-10 was obviously increased in the INHBA-positive expression group. It was suggested that the overexpression of INHBA in CC tissues might have an important relationship with tumor immune response. The reason was that the immune function of the body was impaired when CC occurred. The abnormal expression of Th1/Th2 might, in some cases, stimulate the host immune system, which leads to a systemic anti-tumor immune response, thereby promoting the expression of INHBA [22]. Therefore, INHBA is associated with multiple T-cell biomarkers or common immune checkpoints.

In addition, FIGO staging, differentiation, lymph node metastasis and INHBA are found to be important factors affecting the poor prognosis of patients with CC. The FIGO staging is the most important staging system for CC. A previous study found that FIGO-II patients had a higher survival rate compared with FIGO-IV patients [23]. The degree of differentiation is an important indicator to judge the patient’s condition. The lower the degree of differentiation, the higher the malignancy of the tumor. The ability for invasion and metastasis is another prominent cancer feature, which is related to tumor progression and a reduced survival rate. Tumor invasion and metastasis include a series of events related to the interaction between tumor cells or between tumor cells and the microenvironment, including tumor cells separating from each other, migrating to the extracellular matrix and entering the blood and lymphatic vessels [24]. In the study of endometrial cancer that compared with patients without lymph node metastasis, some scholars found that the overall survival rate of patients with cervical invasion and lymph node vascular invasion was obviously decreased [25]. All these results indicated that it was important to evaluate the prognosis of patients with CC by judging the FIGO stage, differentiation degree, lymph node metastasis and INHBA expression. 

## 5. Conclusions

In general, abnormally high expressions of INHBA in patients with CC were related to pathological characteristics, anti-tumor immune response and survival time, and lead to a poor prognosis. It was speculated that INHBA exerted an important reference role in tumor invasion and clinical prognosis evaluation, which could act as a new target for the anti-tumor treatment of CC. The results of this study provide the relevant theoretical basis for the study of CC progression, and offer some reference for the clinical treatment and prognosis evaluation of CC. However, due to the insufficient sample size and limited research time, specific mechanisms of INHBA in CC is still unclear. In our following basic research, related experiments will be conducted in vivo and in vitro to explore the effect of overexpression or interference of INHBA on the growth, movement, immune response and signal pathway of CC.

## Figures and Tables

**Table 1:** Detection of inhibin beta A (INHBA) expression in cervical cancer (CC) tissues by immunohistochemistry (IHC) (cases (%)).

Groups	Cases	INHBA	
		Positive Expression	Negative Expression
CC tissues	84	57 (67.86)	27 (32.14)
Adjacent tissues	84	24 (28.57)	60 (71.43)
*χ* ^2^		6.478	
*p*		0.011	

**Table 2:** The relationship between expression of INHBA and clinicopathological features in CC (cases (%)).

Groups		Positive Expression (*n* = 57)	Negative Expression (*n* = 27)	*χ* ^2^	*p*
Age (year)	<50	15 (26.32)	6 (22.22)	0.164	0.686
	≥50	42 (73.68)	21 (77.78)		
Federation of Gynecology and Obstetrics (FIGO) stage (%)	Stage Ⅰ	14 (24.56)	14 (51.85)	6.140	0.013
	Stage Ⅱ	43 (75.44)	13 (48.15)		
Tumor size (cm)	≤3	28 (49.12)	10 (37.04)	1.080	0.299
	>3	29 (50.88)	17 (62.96)		
Differentiation degree (%)	Low differentiation	42 (73.68)	13 (48.15)	5.285	0.022
	Medium + high differentiation	15 (26.32)	14 (51.85)		
Number of pregnancies (times)	≤3	19 (33.33)	8 (29.63)	0.115	0.734
	>3	38 (66.67)	19 (70.37)		
Histological type (%)	Adenocarcinoma	6 (10.53)	2 (7.41)	2.610	0.271
	Squamous cell carcinoma	47 (82.46)	20 (74.07)		
	Others	4 (7.02)	5 (18.52)		
Depth of invasion (%)	T1 + T2	20 (35.09)	12 (44.44)	0.680	0.410
	T3 + T4	37 (64.91)	15 (55.56)		
Lymph node metastasis (%)	Yes	38 (66.67)	9 (33.33)	8.260	0.004
	No	19 (33.33)	18 (66.67)		

**Table 3:** The relationship between INHBA expression and the anti-tumor immune response in CC (*x* ± *s*).

Groups	Cases	IFN-γ (pg/mL)	IL-10 (pg/mL)	TNF-α (pg/mL)	IL-2 (pg/mL)
Positive INHBA expression group	57	54.45 ± 18.71	6.87 ± 1.05	145.05 ± 60.26	40.79 ± 13.16
Negative INHBA expression group	27	45.11 ± 10.36	5.03 ± 0.84	108.55 ± 51.49	31.51 ± 12.67
*t*		2.419	7.969	2.711	3.054
*p*		0.018	<0.001	0.009	0.003

**Table 4:** Univariate analysis of clinical prognosis.

Factors		Good Prognosis Group (*n* = 47)	Poor Prognosis Group (*n* = 37)	*χ* ^2^	*p*
Age (year)	<50	8 (17.02)	13 (35.14)	3.623	0.057
	≥50	39 (82.98)	24 (64.86)		
FIGO stage (%)	Stage Ⅰ	22 (46.81)	6 (16.22)	8.719	0.003
	Stage Ⅱ	25 (53.19)	31 (83.78)		
Tumor size (cm)	≤3	21 (44.68)	17 (45.95)	0.013	0.908
	>3	26 (55.32)	20 (54.05)		
Differentiation degree (%)	Low differentiation	37 (78.72)	18 (48.65)	8.284	0.004
	Medium + high differentiation	10 (21.28)	19 (51.35)		
Number of pregnancies (times)	≤3	16 (34.04)	11 (29.73)	0.177	0.674
	>3	31 (65.96)	26 (70.27)		
Histological type (%)	Adenocarcinoma	5 (10.64)	3 (8.11)	2.254	0.324
	Squamous cell carcinoma	35 (74.47)	32 (86.49)		
	Others	7 (14.89)	2 (5.41)		
Depth of invasion (%)	T1 + T2	16 (34.04)	16 (43.24)	0.743	0.389
	T3 + T4	31 (65.96)	21 (83.78)		
Lymph node metastasis (%)	Yes	13 (27.66)	34 (91.89)	34.657	<0.001
	No	34 (72.34)	3 (8.11)		
INHBA expression (%)	Positive	38 (80.85)	9 (24.32)	8.260	0.004
	Negative	9 (19.15)	18 (48.65)		

**Table 5:** Multivariate analysis of the clinical prognosis.

Indicators	B value	Standard Error	Wald Value	*p* Value	OR	95%CI	
						Lower limit	Upper limit
FIGO stage	0.847	0.308	4.519	0.021	3.750	1.240	4.135
Differentiation degree	1.318	0.330	9.311	0.005	4.257	2.236	6.804
Lymph node metastasis	0.963	0.317	6.819	0.014	3.664	1.278	5.366
Expression of INHBA	1.565	0.271	11.853	<0.001	5.158	2.754	7.905

## Data Availability

The datasets used and/or analyzed during the current study are available from the corresponding author on reasonable request.

## References

[1] Gaffney D.K., Hashibe M., Kepka D., Maurer K.A., Werner T.L. (2018). Too many women are dying from cervix cancer: Problems and solutions. Gynecol. Oncol..

[2] Beriwal S., Musunuru H., Pifer P., Mohindra P., Albuquerque K. (2022). Advances in management of locally advanced cervical cancer. Indian J. Med. Res..

[3] Kawagishi-Hotta M., Hasegawa S., Hasebe Y., Inoue Y., Okuno R., Arima M., Iwata Y., Sugiura K., Akamatsu H. (2022). Increase in inhibin beta A/Activin-A expression in the human epidermis and the suppression of epidermal stem/progenitor cell proliferation with aging. J. Derm. Sci..

[4] Liu H., Dai W. (2022). Circular RNA 0000654 facilitates the growth of gastric cancer cells through absorbing microRNA-149-5p to up-regulate inhibin-beta A. Bioengineered.

[5] Tao X., Mei J., Huang Y. (2021). Inhibin subunit beta A promotes cell proliferation and metastasis of breast cancer through Wnt/β-catenin signaling pathway. Bioengineered.

[6] Li X., Yang Z., Xu S., Wang Z., Jin P., Yang X., Zhang Z., Wang Y., Wei X., Fang T. (2019). Targeting INHBA in Ovarian Cancer Cells Suppresses Cancer Xenograft Growth by Attenuating Stromal Fibroblast Activation. Dis. Markers.

[7] Yu Y., Wang W., Lu W., Chen W., Shang A. (2021). Inhibin β-A (INHBA) induces epithelial–mesenchymal transition and accelerates the motility of breast cancer cells by activating the TGF-β signaling pathway. Bioengineered.

[8] Listik E., Horst B., Choi A.S., Lee N.Y., Győrffy B., Mythreye K. (2021). A bioinformatic analysis of the inhibin-betaglycan-endoglin/CD105 network reveals prognostic value in multiple solid tumors. PLoS ONE.

[9] Moreno V., Smith E.A., Piña-Oviedo S. (2021). Fluorescent Immunohistochemistry. Methods Mol Biol..

[10] Li C., Hua K. (2022). Dissecting the Single-Cell Transcriptome Network of Immune Environment Underlying Cervical Premalignant Lesion, Cervical Cancer and Metastatic Lymph Nodes. Front. Immunol..

[11] Peng S., Wang J., Hu P., Zhang W., Li H., Xu L. (2020). INHBA knockdown inhibits proliferation and invasion of nasopharyngeal carcinoma SUNE1 cells in vitro. Int. J. Clin. Exp. Pathol..

[12] Chen Z., Qin L., Peng X., Hu Y., Liu B. (2019). INHBA gene silencing inhibits gastric cancer cell migration and invasion by impeding activation of the TGF-β signaling pathway. J. Cell Physiol..

[13] Li X., Yu W., Liang C., Xu Y., Zhang M., Ding X., Cai X. (2020). INHBA is a prognostic predictor for patients with colon adenocarcinoma. BMC Cancer.

[14] Chen S., Gong Y., Shen Y., Liu Y., Fu Y., Dai Y., Rehman A.U., Tang L., Liu H. (2021). INHBA is a novel mediator regulating cellular senescence and immune evasion in colorectal cancer. J. Cancer.

[15] De Benedetti F., Prencipe G., Bracaglia C., Marasco E., Grom A.A. (2021). Targeting interferon-γ in hyperinflammation: Opportunities and challenges. Nat. Rev. Rheumatol..

[16] Teng F., Cui G., Qian L., Zhao L. (2022). Changes of T Lymphocyte Subsets in Peripheral Blood of Patients with Intermediate and Advanced Cervical Cancer before and after Nimotuzumab Combined with Chemoradiotherapy. Int. Arch. Allergy Immunol..

[17] Alspach E., Lussier D.M., Schreiber R.D. (2019). Interferon γ and Its Important Roles in Promoting and Inhibiting Spontaneous and Therapeutic Cancer Immunity. Cold Spring Harb. Perspect. Biol..

[18] Pol J.G., Caudana P., Paillet J., Piaggio E., Kroemer G. (2019). Effects of interleukin-2 in immunostimulation and immunosuppression. J. Exp. Med..

[19] Meng J., Song J. (2019). Association between interleukin-2, interleukin-10, secretory immunoglobulin A and immunoglobulin G expression in vaginal fluid and human papilloma virus outcome in patients with cervical lesions. Oncol. Lett..

[20] Duvlis S., Dabeski D., Noveski P., Ivkovski L., Plaseska-Karanfilska D. (2020). Association of IL-10 (rs1800872) and IL-4R (rs1805010) polymorphisms with cervical intraepithelial lesions and cervical carcinomas. J. Buon..

[21] Staudacher J.J., Arnold A., Kühl A.A., Pötzsch M., Daum S., Winterfeld M., Berg E., Hummel M., Rau B., Stein U. (2022). Prognostic impact of activin subunit inhibin beta A in gastric and esophageal adenocarcinomas. BMC Cancer.

[22] Zhang S., Jin K., Li T., Zhou M., Yang W. (2022). Comprehensive analysis of INHBA: A biomarker for anti-TGFβ treatment in head and neck cancer. Exp. Biol. Med..

[23] Marnitz S., Tsunoda A.T., Martus P., Vieira M., Junior R.J.A., Nunes J., Budach V., Hertel H., Mustea A., Sehouli J. (2020). Surgical versus clinical staging prior to primary chemoradiation in patients with cervical cancer FIGO stages IIB–IVA: Oncologic results of a prospective randomized international multicenter (Uterus-11) intergroup study. Int. J. Gynecol. Cancer.

[24] Hu S., Li S., Teng D., Yan Y., Lin H., Liu B., Gao Z., Zhu S., Wang Y., Du X. (2021). Analysis of risk factors and prognosis of 253 lymph node metastasis in colorectal cancer patients. BMC Surg..

[25] Li Z., Zhang W., Luo Z., Huang J., Li L. (2021). Clinical study of the clinical characteristics and prognosis of 1219 cases of endometrial cancer with lymph node metastasis. Hum. Exp. Toxicol..

